# Full mouth rehabilitation using advanced concepts with an interdisciplinary approach

**DOI:** 10.6026/973206300210509

**Published:** 2025-03-31

**Authors:** Debkant Jena, Ashtha Arya, Rahul Anand Razdan, Himanshu Sharma, Sitansu Sekhar Das, Raj Rabara

**Affiliations:** 1 Department of Conservative dentistry & Endodontics, Kalinga Institute of Dental Sciences, KIIT University, Bhubaneswar, Odisha, India; 2 Department of Conservative Dentistry and Endodontics, SGT Dental College, Hospital and Research Institute, SGT University, Gurugram, Haryana-122505, India; 3 Department of prosthodontics, crown and Bridge, Index Institute of dental sciences, Indore, Madhya Pradesh, India; 4 Department of Conservative and Endodontics, Modern dental College, Indore, Madhya Pradesh, India; 5 Department of Prosthodontics, Institute Of Dental Sciences, Siksha 'O' Anusandhan Deemed to be University, Bhubaneawar-751003, Odisha, India; 6 Department of Dental Surgeon, Ahemdabad Dental College, Ahemdabad, Gujarat, India

**Keywords:** Full Mouth Rehabilitation, interdisciplinary dental care, advanced dental technologies, treatment success rates, dental specialties, oral health, prosthodontics, periodontology, oral pathology

## Abstract

Full mouth rehabilitation is an interdisciplinary approach integrating advanced technologies to address complex oral health challenges. Therefore, it is of interest to assess knowledge levels, treatment success rates and challenges faced by 500 dental professionals across various specialties using a structured questionnaire. Prosthodontics showed the highest success rate (92%), while oral pathology had the lowest (75%), highlighting the need for targeted training and collaboration. Thus, the importance of interdisciplinary synergy and technology integration to improve full mouth rehabilitation outcomes is highlighted. Hence, continued research and innovation are essential for advancing patient-centered full mouth rehabilitation practices.

## Background:

Full mouth rehabilitation represents a comprehensive and interdisciplinary approach that integrates the expertise of multiple dental specialties to address complex and multifaceted oral health challenges [1, 2- 3]. Unlike isolated dental treatments that focus on singular issues, full mouth rehabilitation takes a holistic view of the oral cavity, recognizing the intricate relationships between its various components [4, 5-6]. By emphasizing the restoration and enhancement of oral function, esthetics and overall health, full mouth rehabilitation aims to not only treat existing problems but also prevent future complications, thereby significantly improving the patient's quality of life [7, 8, 9-10]. The success of full mouth rehabilitation relies heavily on the seamless collaboration of dental specialists, each contributing their unique skills and expertise to create personalized and effective treatment plans [11, 12, 13-14]. Oral surgeons address structural issues such as bone grafting or surgical extractions, while periodontitis focus on gum health and the management of periodontal diseases [15]. Endodontists ensure the integrity of the tooth structure by treating issues related to the pulp and roots, while prosthodontics restore function and esthetics through advanced prosthetic solutions [16]. Restorative dentists play a vital role in repairing and rebuilding teeth to ensure proper occlusion and aesthetics. This interdisciplinary synergy is essential to achieving comprehensive outcomes that cater to the diverse needs of each patient [17]. The scope of full mouth rehabilitation encompasses a broad range of interventions that address the interconnected aspects of the oral system. These include correcting malocclusions to restore proper bite alignment, replacing missing teeth with implants or dentures, repairing damaged teeth with crowns or veneers and managing periodontal health to provide a stable foundation for restorative procedures [18, 19]. Additionally, enhancing oral esthetics, such as improving smile design, contributes significantly to a patient's confidence and overall satisfaction with the treatment. The evolution of dental technologies and techniques has significantly enhanced the potential of full mouth rehabilitation. Tools such as digital diagnostics, 3D imaging and Computer-Aided Design/Computer-Aided Manufacturing technology allow for precise planning and execution of treatments. These advancements enable dental professionals to visualize the final outcome before initiating procedures, reducing errors and improving predictability [20]. Minimally invasive techniques, such as laser dentistry and guided implant surgery, have improved patient comfort and recovery times, while modern prosthetic materials, such as zirconia and lithium disilicate, offer superior durability and esthetics [21]. Together, these innovations have transformed full mouth rehabilitation into a patient-centered, efficient and effective process. Despite these advancements, full mouth rehabilitation remains a challenging endeavor due to its complexity and the need for a tailored approach for each patient. Dental professionals must navigate various challenges, including patient-specific anatomical and functional limitations, pre-existing conditions and the integration of multiple specialties into a cohesive treatment plan [22]. Therefore, it is of interest to assess knowledge levels, treatment success rates and challenges faced by 500 dental professionals across various specialties using a structured questionnaire.

## Methodology:

The study was designed as a cross-sectional survey aimed at evaluating treatment success rates, knowledge levels and challenges faced across various dental specialties. A total of 500 dental professionals participated, representing a diverse sample from key specialties, including Oral Surgery, Periodontology, Conservative Dentistry, Endodontics, Oral Pathology, Prosthodontics and Restorative Dentistry. This diverse representation ensured a comprehensive understanding of interdisciplinary trends and specialty-specific performance metrics. Data collection was carried out using a structured questionnaire, meticulously designed to capture quantitative and qualitative insights. The questionnaire included sections on knowledge scores, treatment success rates and challenges encountered in clinical practice, enabling a holistic assessment of the professionals' experiences and expertise. For data analysis, both descriptive and inferential statistical methods were employed, using SPSS version [25]. Descriptive statistics provided an overview of the mean scores and percentages across variables, while inferential techniques allowed for comparisons and correlations between specialties. This rigorous analytical approach ensured robust findings, highlighting key patterns and relationships in the data. By integrating insights from structured data collection and advanced statistical tools, the study offered valuable evidence on the current state of dental specialties, underscoring the need for targeted improvements and interdisciplinary collaboration.

## Results:

The comparative analysis of treatment success rates across dental specialties highlights significant insights into clinical performance. Prosthodontics leads with the highest success rate of 92%, showcasing its advanced techniques, precise treatment planning and successful patient outcomes. This indicates a strong foundation in clinical knowledge and application. Oral Surgery, Endodontics and Restorative Dentistry also demonstrate high success rates (ranging from 87% to 90%), reflecting their effective approaches to managing complex cases and delivering reliable results. In contrast, Oral Pathology records the lowest success rate at 75%, which could be attributed to the inherent complexity of diagnosing and managing rare or challenging pathological conditions. Periodontology and Conservative Dentistry exhibit moderate success rates (85% and 88%, respectively), suggesting room for further innovation and interdisciplinary collaboration. Overall, the data underscores the importance of leveraging strengths across specialties to address specific challenges. Knowledge sharing targeted training programs and team-based approaches can significantly enhance treatment success rates, particularly in fields with higher challenges. This holistic, interdisciplinary effort is key to achieving consistent and improved outcomes in dental care (Table 1 and Figure 1 (see PDF)).

## Discussion:

Dental specialties play a critical role in delivering comprehensive oral healthcare, with each specialty contributing unique expertise to patient care. Advances in techniques, materials and interdisciplinary collaboration have significantly influenced treatment success rates. Numerous studies have highlighted the importance of specialized knowledge, skill enhancement and evidence-based practice in improving patient outcomes across various dental fields [23]. In the current study, the comparative analysis of treatment success rates, knowledge scores and challenges faced across dental specialties provided valuable insights into clinical performance. The findings align with previous literature emphasizing the need for targeted training and interdisciplinary collaboration to optimize patient outcomes. Several studies have reported similar trends in treatment success rates among dental specialties. According to Venkatesan *et al.* [23] prosthodontics consistently demonstrates high success rates due to advancements in digital workflows, precision in treatment planning and material innovations. Our study aligns with these findings, reporting the highest success rate of 92% in prosthodontics, reinforcing its well-established clinical efficacy [24]. Conversely, oral pathology presented the lowest treatment success rate (75%) in our study. This suggests a need for enhanced diagnostic tools, early detection strategies and interdisciplinary collaboration to improve treatment outcomes in oral pathology. The success rates in oral surgery, endodontics and restorative dentistry (87%-90%) reflect findings from previous research, which highlighted the impact of evidence-based protocols, technological integration and case selection on clinical success. These specialties have leveraged advanced imaging techniques and minimally invasive approaches to achieve favourable outcomes [25]. Our study's findings on knowledge scores are consistent with literature emphasizing the role of continuous education and professional development in enhancing specialty-specific expertise [26]. Periodontology and conservative dentistry showed moderate knowledge scores (78% and 80%, respectively), aligning with earlier review which suggested the need for more evidence-based training and exposure to emerging technologies to boost expertise and performance [27]. Er-YAG laser surface treatment significantly enhances zirconia bonding, making it a valuable technique in full mouth rehabilitation by improving restoration longevity and clinical success. Given the interdisciplinary nature of FMR, integrating advanced surface treatments ensures stronger adhesion, enhancing prosthodontic outcomes and overall treatment durability. The findings suggest that Er-YAG laser and sandblasting are both effective, but laser treatment offers a more controlled approach, minimizing surface damage. Incorporating these methods into full mouth rehabilitation protocols can optimize prosthetic stability, supporting long-term patient satisfaction and interdisciplinary collaboration in complex [28].

Challenges faced by dental professionals varied across specialties, with oral pathology reporting the highest challenge rate (50%), which identified the limited availability of diagnostic resources and the complexity of pathological conditions as major barriers. Similarly, periodontology faced significant challenges (40%), highlighted the difficulty in achieving long-term periodontal stability and patient compliance. Prosthodontics reported the lowest challenge rate (28%), which aligns with its high success rate and knowledge scores. This reinforces the importance of systematic treatment protocols and technological integration in overcoming clinical hurdles. Orthodontic failure in full mouth rehabilitation often stems from inadequate treatment planning, occlusal discrepancies and patient compliance issues. The findings underscore the need for continuous professional development, targeted training programs and interdisciplinary collaboration to address specialty-specific challenges. Knowledge sharing and team-based approaches can help enhance treatment success rates across all specialties. Efforts should prioritize the implementation of advanced diagnostic tools and evidence-based protocols in oral pathology to enhance accuracy and efficiency in diagnosis and treatment. Promoting interdisciplinary case discussions is essential for improving patient outcomes in periodontology and conservative dentistry by fostering collaborative approaches and shared expertise. Additionally, encouraging lifelong learning and the adoption of emerging technologies is vital for maintaining high success rates in prosthodontics and endodontics, ensuring practitioners stay updated with advancements and deliver optimal care.

## Conclusion:

A comprehensive evaluation of dental specialties, highlighting key areas for improvement and reinforcing the importance of specialized knowledge, clinical expertise and interdisciplinary teamwork in optimizing patient care is shown. Future research should explore innovative training interventions and their impact on clinical outcomes across diverse dental fields.

## Figures and Tables

**Table 1:** Knowledge, success rates and challenges in full mouth rehabilitation across dental specialties

**Specialty**	**Knowledge Score (%)**	**Treatment Success (%)**	**Challenges Faced (%)**
Oral Surgery	85	90	30
Periodontology	78	85	40
Conservative	80	88	35
Endodontics	82	89	32
Oral Pathology	70	75	50
Prosthodontics	88	92	28
Restorative	84	87	33
Orthodontics	81	86	37
